# Supplementary material for: Spurious North Tropical Atlantic precursors to El Niño
Source: Nat Commun. 2021 May 25;12:3096. doi: 10.1038/s41467-021-23411-6 (PMC8149707; doi:10.1038/s41467-021-23411-6)
Supplement: Supplementary file 1 — Supplementary information [file 41467_2021_23411_MOESM1_ESM.pdf]

Spurious North Tropical Atlantic precursors to El Niño

Supplementary Information

Wenjun Zhang[1], Feng Jiang[1], Malte F. Stuecker[2], Fei-Fei Jin[3], Axel Timmermann[4,5]

[1]Key Laboratory of Meteorological Disaster of Ministry of Education (KLME), Nanjing University of Information Science and Technology, Nanjing, China

[2]Department of Oceanography & International Pacific Research Center (IPRC), School of Ocean and Earth Science and Technology (SOEST), University of Hawai'i at Mānoa, Honolulu, HI, USA

[3]Department of Atmospheric Sciences, School of Ocean and Earth Science and Technology (SOEST), University of Hawai'i at Mānoa, Honolulu, HI, USA

[4]Institute for Basic Science, Center for Climate Physics, Busan, South Korea

[5]Pusan National University, Busan, South Korea

Corresponding author address:

Dr. Wenjun Zhang
School of Atmospheric Sciences, Nanjing University of Information Science and Technology, Nanjing 210044, China.
E-mail: zhangwj@nuist.edu.cn

Prof. Fei-Fei Jin
Department of Atmospheric Sciences, SOEST,
University of Hawai'i at Mānoa, Honolulu, HI 96822, USA
E-mail: jff@hawaii.edu

Supplementary Discussion

Here we demonstrate that complicated ENSO cycle features may not affect the qualitative relationship of North Tropical Atlantic (NTA) sea surface temperature (SST) with following ENSO based on our ENSO-forced NTA model. Three experiments are performed by using different ENSO cycles as in the case of a 4-yr cycle. A perfectly 4-yr sinusoidal ENSO cycle is prescribed in EXP1 for reference (black line in Supplementary Fig. 4a). Then a possibly more realistic 4-yr ENSO cycle comprising 1-yr El Niño and 3-yr La Niña is prescribed in EXP2 (red line in Supplementary Fig. 4a). Besides, the possible interference from the amplitude asymmetry of El Niño and La Niña episodes is also examined in EXP3 by multiplying El Niño amplitude by a factor of 2 in EXP1 (blue line in Supplementary Fig. 4a). Using the parameters estimated in Supplementary Table 1, we force the NTA model with different ENSO evolutions for 25 ENSO cycles (1200 months) and then derive the respective NTA time series. Qualitative ENSO-NTA lead-lag relationships can be obtained under the consideration of these ENSO irregular behaviors including duration and amplitude asymmetries, despite slight differences in the respective correlation coefficients (Supplementary Fig. 4b).

Supplementary Table 1. Model parameters estimated using the monthly observed NTA SST and Nino3.4 index

Parameters	unit：/month
λ_0	0.13
D	0.38
φ_D	0.49
α_0	-0.10
A	0.33
φ_A	-0.25

Supplementary Table 2. 46 CMIP6 models are used in pi-control simulations and the availability of SSP2-4.5 and SSP5-8.5 simulations.

Models	Institution, Country	SSP2-4.5	SSP5-8.5
ACCESS-CM2	CSIRO-ARCCSS, Australia	yes	yes
BCC-CSM2-MR	BCC, China	yes	yes
BCC-ESM1	BCC, China		
CAMS-CSM1-0	CAMS, China	yes	yes
CanESM5	CCCma, Canada	yes	yes
CanESM5-CanOE	CCCma, Canada		
CESM2	NCAR, USA	yes	
CESM2-FV2	NCAR, USA		
CESM2-WACCM	NCAR, USA	yes	yes
CIESM	THU, China	yes	yes
CNRM-CM6-1	CNRM-CERFACS, France	yes	yes
CNRM-CM6-1-HR	CNRM-CERFACS, France	yes	yes
CNRM-ESM2-1	CNRM-CERFACS, France	yes	yes
E3SM-1-0	E3SM-Project		
E3SM-1-1-ECA	E3SM-Project		
E3SM-1-1	E3SM-Project		
EC-Earth3	EC-Earth-Consortium	yes	yes
EC-Earth3-LR	EC-Earth-Consortium		
EC-Earth3-Veg	EC-Earth-Consortium		yes
EC-Earth3-Veg-LR	EC-Earth-Consortium		
FGOALS-f3-L	CAS, China	yes	yes
FGOALS-g3	CAS, China	yes	yes
FIO-ESM-2-0	FIO-QLNM, China	yes	yes
GFDL-CM4	NOAA-GFDL, USA	yes	yes
GFDL-ESM4	NOAA-GFDL, USA		yes
GISS-E2-1-G-CC	NASA-GISS, USA		
GISS-E2-1-G	NASA-GISS, USA	yes	yes
GISS-E2-1-H	NASA-GISS, USA		
GISS-E2-2-G	NASA-GISS, USA		
HadGEM3-GC31-LL	MOHC, UK		
IITM-ESM	CCCR-IITM, India		
INM-CM4-8	INM, Russia	yes	yes
IPSL-CM6A-LR	IPSL, France		yes
MCM-UA-1-0	UA, USA	yes	
MIROC-ES2L	MIROC, Japan	yes	yes
MIROC6	MIROC, Japan		yes
MPI-ESM-1-2-HAM	MPI-M, Germany		
MPI-ESM1-2-HR	MPI-M, Germany		yes
MPI-ESM1-2-LR	MPI-M, Germany	yes	yes

MRI-ESM2-0	MRI, Japan	yes	yes
NESM3	NUIST, China	yes	yes
NorESM1-F	NCC, Norway		
NorESM2-LM	NCC, Norway	yes	yes
NorESM2-MM	NCC, Norway	yes	yes
SAM0-UNICON	SNU, Republic of Korea		
UKESM1-0-LL	MOHC, UK	yes	yes

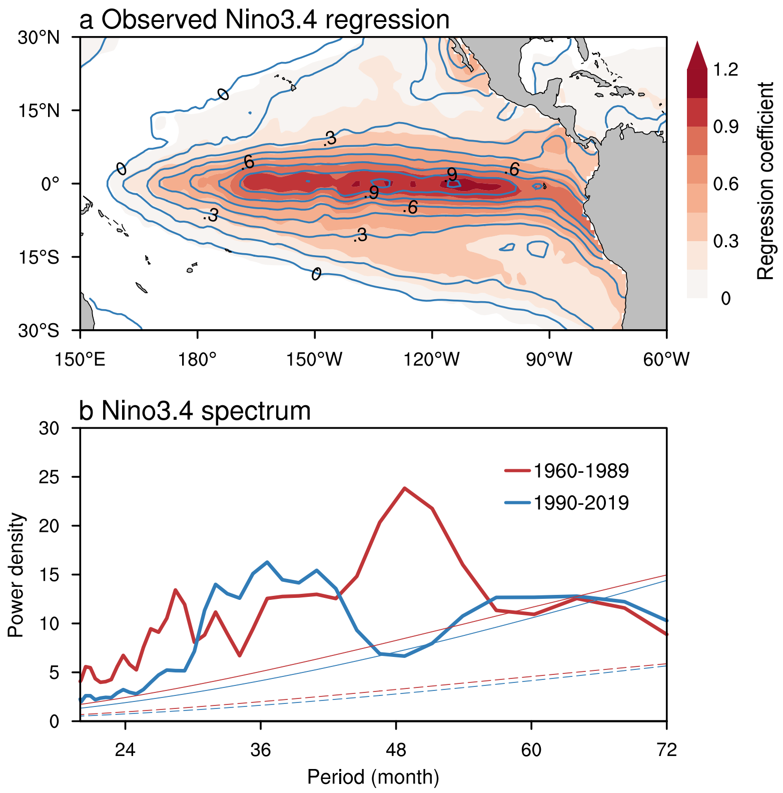

Supplementary Figure 1. ENSO regime shift around 1990. a Regression of sea surface temperature anomalies upon the Niño3.4 index for the periods of 1960-1989 (shading) and 1990-2019 (contours with interval: 0.3°C). **b** Multi-Taper-Method power spectra for the periods of 1960-1989 (solid thick red) and 1990-2019 (solid thick blue). The averaged AR(1) null hypothesis is displayed by a dashed thin line and the 95% confidence level is indicated by a solid thin line.

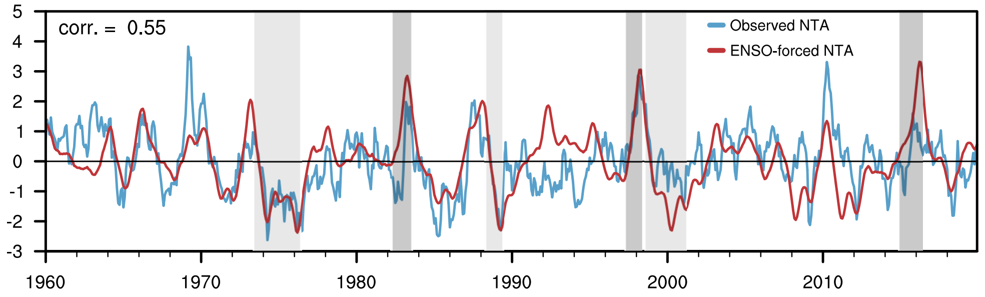

Supplementary Figure 2. Monthly standardized NTA SST anomaly time series. Grey rectangles indicate the duration (±0.5°C threshold of the Niño3.4 index) of the three strongest El Niño and La Niña events (determined by the boreal winter Niño3.4 index) for observation (blue) and reconstruction (red).

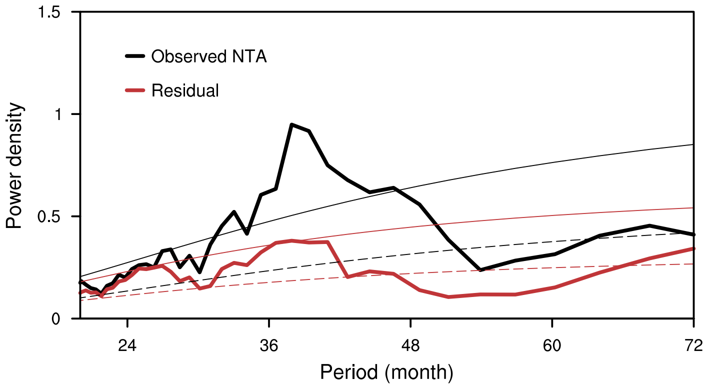

Supplementary Figure 3. MTM power spectra for the observed NTA SST anomaly (solid red) and the associated residual (solid black). The residual is defined as difference between the observation and reconstruction. The averaged AR(1) null hypothesis is displayed by a dashed thin line and the 95% confidence level is indicated by a solid thin line.

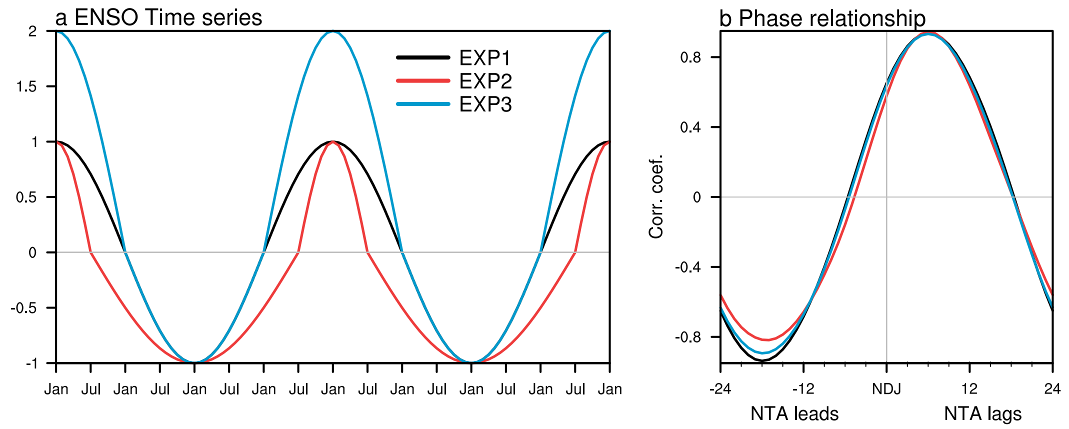

Supplementary Figure 4. Impacts of complicated ENSO cycle features on the ENSO-NTA SST relationship. a Idealized ENSO time series (°C) specified in EXP1 (black line), EXP2 (red line) and EXP3 (blue line). **b** Lead-lagged correlation of the boreal winter ENSO with NTA time series for EXP1 (black line), EXP2 (red line) and EXP3 (blue line). A nine-point smoothing is applied in **b**.

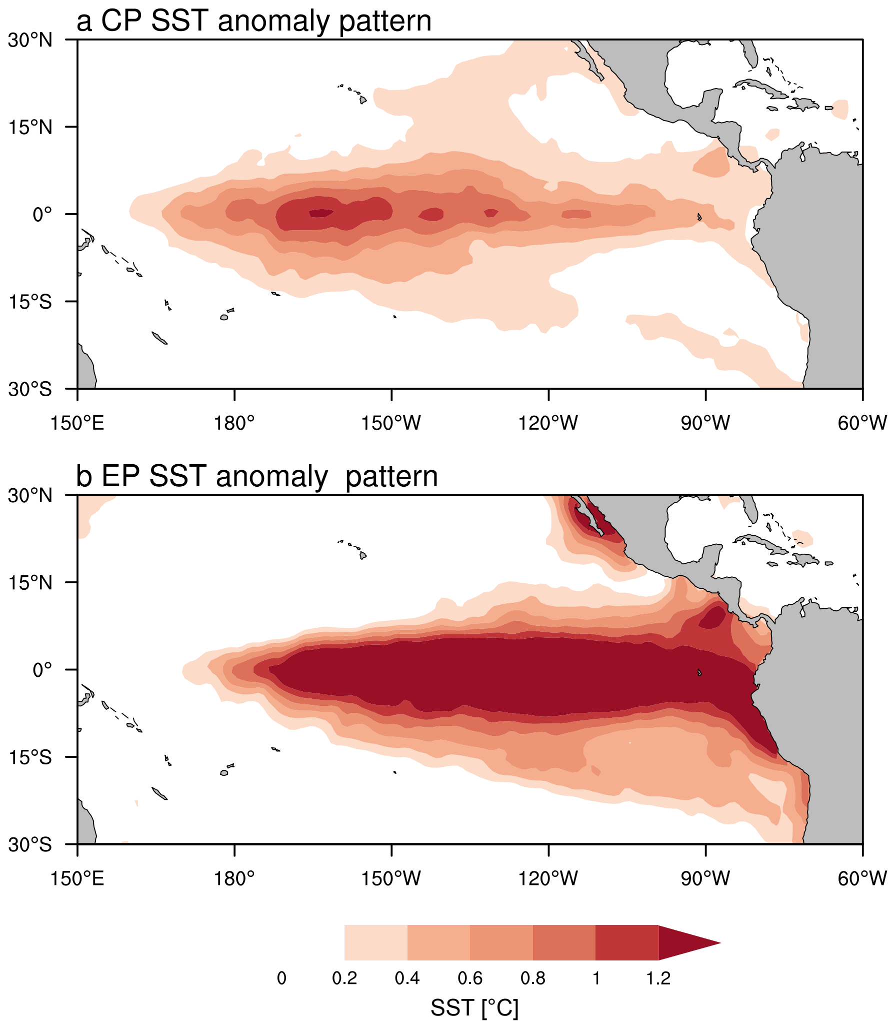

Supplementary Figure 5. ENSO-related SST anomaly pattern. Prescribed sea surface temperature anomaly (°C) forcing associated with a CP El Niño and b EP El Niño composites.

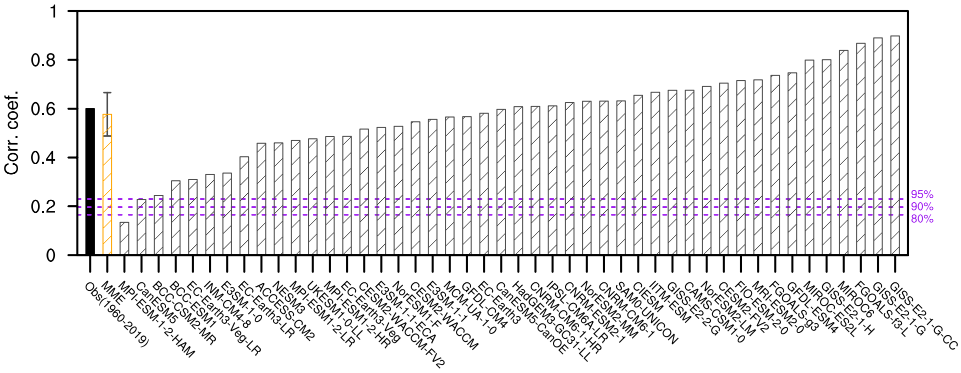

Supplementary Figure 6. Lead correlation of boreal winter Niño3.4 index with the following spring NTA SST anomaly for 46 CMIP6 models and observations as a reference. The error bar for the multi-model ensemble (MME) mean corresponds to one standard deviation. The models are ranked by the ENSO/North Tropical Atlantic (NTA) correlation coefficients in an ascending order. The dashed purple lines represent the 80%, 90% and 95% confidence levels.

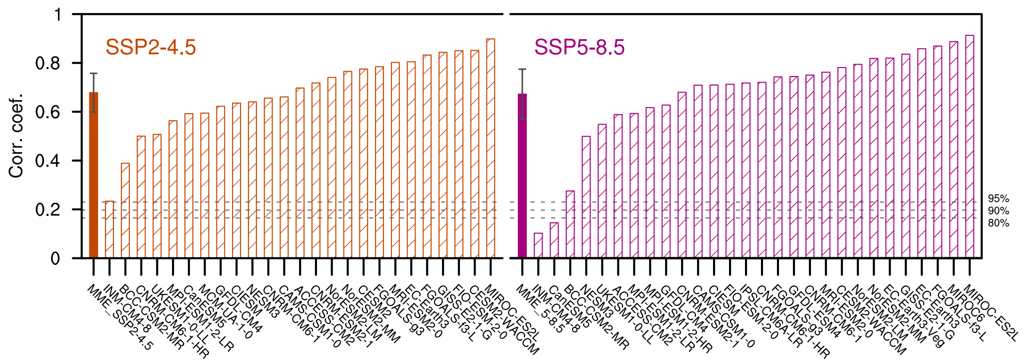

Supplementary Figure 7. Lead correlation of boreal winter Niño3.4 index with the following spring NTA SST anomaly for 25 models from the SSP2-4.5 (orange) and 28 models from the SSP5-8.5 (purple) scenarios. Error bars in the multi-model ensemble (MME) mean correspond to one standard deviation. The models are ranked by the ENSO/ North Tropical Atlantic (NTA) correlation coefficients in an ascending order. The dashed gray lines represent the 80%, 90% and 95% confidence levels.

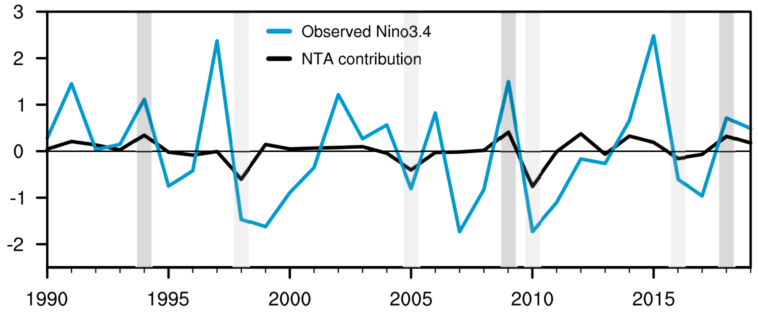

Supplementary Figure 8. ENSO events with appreciable NTA contributions. Observed (blue line) and hindcasted (black line) boreal winter Niño3.4 index (°C) using the previous spring North Tropical Atlantic (NTA) sea surface temperature (SST) after the 1990s. Light and dark grey rectangles indicate the El Niño and La Niña years with appreciable NTA contributions (see Methods), respectively.

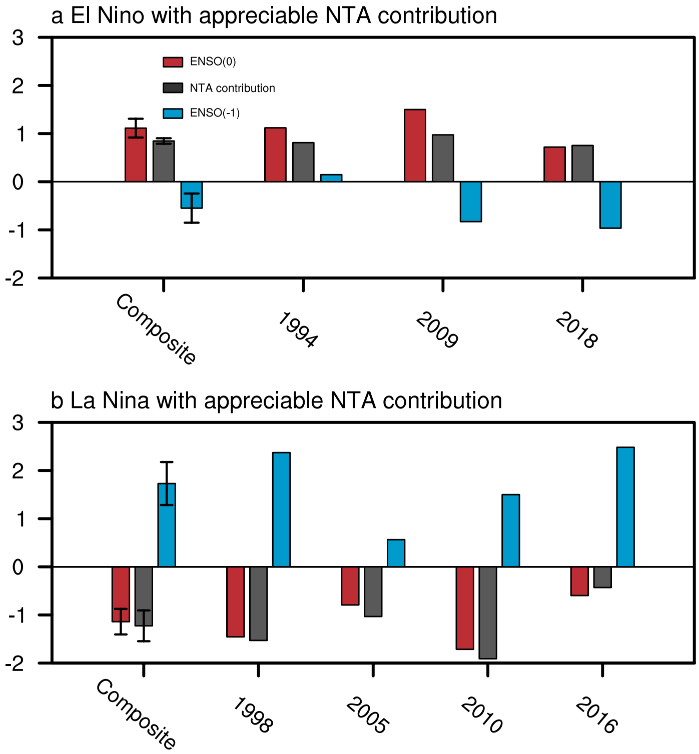

Supplementary Figure 9. Preconditions in the tropical Pacific for ENSO events with appreciable NTA contributions. a El Niño and **b** La Niña events with appreciable North Tropical Atlantic (NTA) contributions after the 1990s and their composites with error bars corresponding to one standard deviation. Red bars denote the winter Niño3.4 index (°C), gray bars the NTA contribution (°C) and blue bars the winter Niño3.4 index (°C) of the previous year.

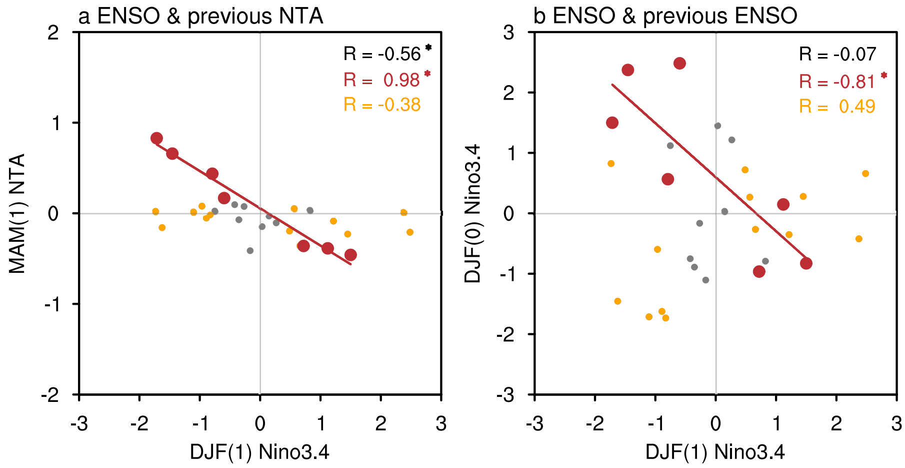

Supplementary Figure 10. Scatterplots of ENSO with its preconditions in the NTA and tropical Pacific. Relationship between the boreal winter Niño3.4 index with **a** the previous spring North Tropical Atlantic (NTA) sea surface temperature (SST) anomalies and **b** the previous winter Niño3.4 index after the 1990s. Red dots in (**a-b**) denote the ENSO years with appreciable NTA contributions, orange dots the other ENSO years, and gray dots the residual ENSO-neutral years. The correlation coefficients (R) for all dots, red dots, and orange dots are shown. Asterisk indicates that the correlation coefficient is statistically significant at the 95% confidence level. The linear fit for the red dots is also displayed.
